# Comprehensive analysis of BTN3A1 in cancers: mining of omics data and validation in patient samples and cellular models

**DOI:** 10.1002/2211-5463.13256

**Published:** 2021-08-04

**Authors:** Fan Liang, Chen Zhang, Hua Guo, San‐Hui Gao, Fu‐Ying Yang, Guang‐Biao Zhou, Gui‐Zhen Wang

**Affiliations:** ^1^ State Key Laboratory of Membrane Biology Institute of Zoology Chinese Academy of Sciences & University of Chinese Academy of Sciences Beijing China; ^2^ State Key Laboratory of Molecular Oncology National Cancer Center/National Clinical Research Center for Cancer/Cancer Hospital Chinese Academy of Medical Sciences and Peking Union Medical College Beijing China

**Keywords:** breast cancer, BTN3A1, immune microenvironment, non‐small cell lung cancer

## Abstract

*Butyrophilin 3A1* (*BTN3A1*), a major histocompatibility complex‐associated gene that encodes a membrane protein with two extracellular immunoglobulin domains and an intracellular B30.2 domain, is critical in T‐cell activation and adaptive immune response. Here, the expression of *BTN3A1* in cancers was analyzed in eight databases comprising 86 733 patients of 33 cancers, and the findings were validated in patient samples and cell models. We showed that *BTN3A1* was expressed in most cancers, and its expression level was strongly correlated with clinical outcome of 13 cancers. Mutations of *BTN3A1* were detected, and the mutations were distributed throughout the entire gene. Gene set enrichment analysis showed that *BTN3A1* co‐expression genes and interacting proteins were enriched in immune regulation‐related pathways. BTN3A1 was associated with tumor‐infiltrating immune cells and was co‐expressed with multiple immune checkpoints in patients with breast cancer (BRCA) and non‐small cell lung cancer (NSCLC). We reported that BTN3A1 was downregulated in 46 of 65 (70.8%) NSCLCs, and its expression level was inversely associated with clinical outcome of the patients. BTN3A1 in tumor samples was lower than in counterpart normal tissues in 31 of 38 (81.6%) BRCAs. Bioinformatics analyses showed that *BTN3A1* could be a target gene of transcription factor Spi‐1 proto‐oncogene (SPI1), and our ‘wet’ experiments showed that ectopic expression of SPI1 upregulated, whereas silencing of SPI1 downregulated, BTN3A1 expression in cells. These results suggest that BTN3A1 may function as a tumor suppressor and may serve as a potential prognostic biomarker in NSCLCs and BRCAs.

## Graphical abstract

*BTN3A1* was altered in most cancers and was downregulated and positively associated with prognosis of patients with non‐small cell lung cancer and breast cancer. BTN3A1 was correlated with immune cell infiltration and immune checkpoints' expression and was a target gene of transcription factor SPI1. It may function as a tumor suppressor and plays a role in carcinogenesis.

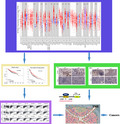

**Abbreviations**
BLCA: bladder urothelial carcinoma
BRCA: breast cancer
BTN3A1: butyrophilin 3A1
CHOL: cholangiocarcinoma
COSMIC: Catalogue of Somatic Mutations in Cancer
CTLA‐4: cytotoxic T‐lymphocyte antigen‐4
ESCA: esophageal carcinoma
FDR: false discovery rate
GO: Gene Ontology
GSEA: gene set enrichment analysis
HNSC: head and neck squamous cell carcinoma
Ig: immunoglobulin
IHC: immunohistochemistry
KICH: kidney chromophobe
KIRC: kidney renal clear cell carcinoma
KIRP: kidney renal papillary cell carcinoma
LGG: brain lower grade glioma
LIHC: liver hepatocellular carcinoma
LUAD: lung adenocarcinoma
LUSC: lung squamous cell carcinoma
MDSCs: myeloid‐derived suppressor cells
MESO: mesothelioma
NSCLC: non‐small cell lung cancer
PD‐1: programmed cell death 1
PD‐L1: programmed cell death 1 ligand
PPI: protein–protein interaction
PRAD: prostate adenocarcinoma
qRT‐PCR: quantitative reverse transcription‐polymerase chain reaction
READ: rectum adenocarcinoma
SARC: sarcoma
SKCM: skin cutaneous melanoma
SPI1: Spi‐1 proto‐oncogene
STAD: stomach adenocarcinoma
TCGA: The Cancer Genome Atlas
TGCT: testicular germ cell tumors
TIMER: Tumor Immune Estimation Resource
TSS: transcription start site
UCEC: uterine corpus endometrial carcinoma

Members of the B7:CD28 family play key roles in regulating T‐cell activation and in cancer development and progression [1, 2]. Blocking the immune checkpoints including programmed cell death 1 (PD‐1)/programmed cell death 1 ligand (PD‐L1) and cytotoxic T‐lymphocyte antigen‐4 (CTLA‐4) significantly prolongs the overall survival of 20–30% patients of most subtypes of cancers [3, 4]. However, immune checkpoint inhibitors do not provide a long‐term benefit to the majority of cancer patients, and there is still an urgent need to explore new targets for the development of cancer immunotherapy [5].

Butyrophilin (BTN) family belongs to the immunoglobulin (Ig) superfamily, whose family members have cytoplasmic and extracellular regions. The cytoplasmic region contains B30.2 domain, which is also known as PRY/SPRY domain and has an important role in protein–protein interaction (PPI) [6, 7], mediating its interaction with proteins including major histocompatibility complex family proteins and proteins associated with Interleukin‐1B secretion [8, 9, 10]. The extracellular domain of BTN is similar to that of the B7 family proteins, suggesting that BTN family members may also have immunomodulatory properties. Recent studies show that BTN family members regulate the immune responses of T cells, especially the γδ T cells [11], and targeting these molecules is emerging as an attractive strategy for cancer immunotherapy [12]. Butyrophilin 3A1 (*BTN3A1*), also known as *CD277*, is a member of the BTN3A subfamily. It can be found in stressed cells, malignant cells, and immune cells such as T cells, natural killer cells, and monocytes [12]. *BTN3A1* harbors a B30.2 domain in the cytoplasmic region and B7‐like domain in the extracellular region. The most well‐known function of *BTN3A1* is mediating the activation of vγ9vδ2 T cells, using its B30.2 domain to bind the phosphorylated antigens to stimulate the cells [13, 14, 15]. *BTN3A1* can inhibit tumor responsive αβ T‐cell receptor activation by preventing N‐glycosylated CD45 from dissociation from the immune synapse [16]. In addition, activation of *BTN3A1* can significantly enhance the TCR‐induced T‐cell proliferation and cytokine secretion [17]. It is reported that *BTN3A1* plays a critical role in cytosolic DNA‐ or RNA‐mediated type I interferon (IFN) responses through promoting interferon regulatory factor 3 phosphorylation and IFN‐β secretion [18].

Although the significance of the *BTN3A1* in the activation of γδ T cells has been documented in previous studies, its role in carcinogenesis remains unclear. In this study, 8 databases comprising 33 cancer types and 86 733 cases were accessed to analyze the expression, mutation, enrichment of related signal pathways of *BTN3A1*, and its relationship with immune cell infiltration and the prognosis of cancer patients. The findings of bioinformatics analyses were validated by experiments in patient samples and cell models. Our results demonstrated that *BTN3A1* was perturbed in cancers and this gene might have an important role in cancer development and progression.

## Materials and methods

### The expression of *BTN3A1* in human cancers

The expression of *BTN3A1* in tumor and counterpart normal tissues of various types of cancers was analyzed using the datasets of Oncomine database (https://www.oncomine.org/resource/login.html) [19], The Cancer Genome Atlas (TCGA; http://cbioportal.org), and Tumor Immune Estimation Resource (TIMER; https://cistrome.shinyapps.io/timer/) [20].

### Pan‐cancer survival analysis

The potential association between the expression level of *BTN3A1* and the clinical outcome of cancers was analyzed by using the online survival analysis Software (the Kaplan–Meier Plotter; http://kmplot.com/analysis/) [21] and the Gene Expression Profiling Interactive Analysis (gepia.cancer‐pku.cn/) [22].

### The mutation analysis of *BTN3A1* in human cancers

cBio Cancer Genomics Portal (http://cbioportal.org) [23] and Catalogue of Somatic Mutations in Cancer (COSMIC) database (V92) (https://cancer.sanger.ac.uk/cosmic/) [24] were used to analyze mutation and copy number variation of *BTN3A1* in Pan‐cancer.

### Gene set enrichment analysis

The biological process categories of Gene Ontology (GO) for the gene set enrichment analysis (GSEA) in Linkedomics database (http://www.linkedomics.org/login.php) were analyzed to determine the enriched pathways that are in correlation with *BTN3A1* [25]. The rank criterion was false discovery rate (FDR) ≤ 0.05, and 10 000 simulations were performed.

### *BTN3A1* and the immune cell infiltration

TIMER and TIMER2.0 were utilized to analyze the correlation between *BTN3A1* expression level and the abundance of immune infiltrates and tumor purity [26]. GEPIA was employed to analyze the correlation between the expression of *BTN3A1* and immune checkpoints.

### Potential regulatory mechanism of *BTN3A1*

The transcription factors that may regulate *BTN3A1* expression level were predicted by gcbi online software (https://www.gcbi.com.cn), Cistrome Data Browser (http://cistrome.org/db/) [27] and GeneCards (https://www.genecards.org) [28]. The PPI network of BTN3A1 was validated by String Database (V11.0; https://string‐db.org/) [29].

### Patient samples

The study was approved by the research ethics committee of Chinese Academy of Medical Sciences Cancer Institute and Hospital. All samples were collected with written informed consent, and the study methodologies conformed to the guidelines set by the Declaration of Helsinki. The diagnosis of non‐small cell lung cancer (NSCLC) and breast cancer (BRCA) was confirmed by at least two pathologists. NSCLC and BRCA tissue samples were obtained at the time of surgery and quickly frozen in liquid nitrogen. A tissue microarray containing tumor tissues and adjacent nontumor tissues isolated from 30 BRCA patients was purchased from Shanghai Outdo Biotech (Shanghai, China). The baseline demographic characteristics of patients with NSCLC and BRCA are listed in Tables 1 and 2, respectively.

**Table 1:** Baseline demographic characteristics of the 65 NSCLC patients.

Variable	Cases, *n* (%)	BTN3A1 expression		
		High, *n* (%)	Low, *n* (%)	*P* values^a^
Total	65	19 (29.2)	46 (70.8)	
Age				
≤ 60	37 (56.9)	12 (32.4)	25 (67.6)	0.51
> 60	28 (43.1)	7 (25)	21 (75)	
Gender				
Male	45 (69.2)	14 (31.1)	31 (68.9)	0.62
Female	20 (30.8)	5 (25)	15 (75)	
Tobacco smoke				
Smoker	35 (53.8)	10 (28.6)	25 (71.4)	0.90
Nonsmoker	30 (46.2)	9 (30)	21 (70)	
Stage				
I	23 (35.4)	8(34.8)	15 (65.2)	0.20
II	11 (16.9)	1 (9.1)	10 (90.9)	
III–IV	29 (44.6)	10 (34.5)	19 (65.5)	
Not recorded	2 (3.1)	0 (0)	2 (100)	

^a^ *P* values were calculated using a two‐sided Fisher's exact test.

**Table 2:** Baseline demographic characteristics of the 38 BRCA patients.

Variable	No. of cases (%)	BTN3A1 expression		
		High, *n* (%)	Low, *n* (%)	*P* values^a^
Total	38	7 (18.4)	31 (81.6)	
Age				
≤ 50	20 (52.6)	4 (20)	16 (80)	0.79
> 50	18 (47.4)	3 (16.7)	15 (83.3)	
Stage				
I	5 (13.2)	1 (20)	4 (80)	0.51
II	23 (60.5)	3 (13)	20 (87)	
III–IV	10 (26.3)	3 (30)	7 (70)	

^a^ *P* values were calculated using a two‐sided Fisher's exact test.

### Immunohistochemistry assay

Immunohistochemistry (IHC) assay was performed to test BTN3A1 expression in NSCLC and BRCA patient samples. Tissue specimens were fixed in formalin and embedded in paraffin, subjected to a heat‐induced epitope retrieval step in citrate buffer solution after deparaffinized through xylene and graded alcohol. The sections were then blocked with 5% BSA for 30 min and incubated with anti‐BTN3A1 antibody (#25221‐1‐AP; Proteintech, Rosemont, IL, USA; 1 : 50) at 4 °C overnight, followed by incubation with HRP‐labeled Goat Anti‐Mouse IgG antibodies for 90 min at 37 °C. Immunoreactions were detected using 3,3′‐diaminobenzidine (DAB, Zhongshan Golden Bridge Biotechnology Co., Ltd., Beijing, China) and hematoxylin. The immunoreactivity score was calculated as IRS (0–12) = RP (0–4) × SI (0–3), where RP is the percentage of staining‐positive cells and SI is staining intensity.

### Luciferase assay

Human breast cancer cell line MCF‐7 and NSCLC line H520 were cultured in Dulbecco's modified Eagle medium supplemented with 10% fetal bovine serum (Gibco, Grand Island, NY, USA). The cells were transfected with plasmids containing *BTN3A1* promoter‐driven luciferase, constructs containing Spi‐1 proto‐oncogene (*SPI1*) coding sequence, or small interfering RNAs (siRNAs) (Table 3). Total RNA of the cells was extracted with TRIZOL reagent (Invitrogen, Frederick, MD, USA), and the expression of interested genes was tested by quantitative reverse transcription‐polymerase chain reaction (qRT‐PCR) using primers listed in Table 3. Luciferase activity was measured using the Dual‐luciferase reporter assay system (Promega, Madison, WI, USA).

**Table 3:** Sequences of primers and siRNAs used in this study.

Target	Forward primer (5′–3′)	Reverse primer (5′–3′)
ChIP		
*BTN3A1*	TGTCAAGGTTGCCTCATGGTC	TGTGACTGGGGCTGATGCAAT
siRNA		
siNC	UUCUCCGAACGUGUCACGUTT	ACGUGACACGUUCGGAGAATT
si*SPI1*‐1	AGCGAUCACUAUUGGGAUUTT	AAUCCCAAUAGUGAUCGCUTT
si*SPI1*‐2	UCGUAAGUAACCAAGUCAUTT	AUGACUUGGUUACUUACGATT
qPCR		
*BTN3A1*	AAAGCACAAGAGTGAAGCTCC	TCCTCTGGTCCTCAGAAACAA

### Chromatin immunoprecipitation

A total of 2 × 10^7^ cells were fixed with 1% formaldehyde for 10 min at room temperature and then stopped by 0.125 m Glycine. The protein‐bound chromatin was fragmented by sonication and divided equally into two parts for ChIP assay or control. 95% of the sonication‐treated chromatin was immunoprecipitated at 4 °C overnight with an anti‐SPI1 antibody (#2258S; Cell Signaling Technology, Boston, MA, USA, 1 : 100) for ChIP or normal rabbit IgG (#2729; Cell Signaling Technology, 1 : 100) as a control while the remaining 5% was termed ‘Input’. Protein A/G PLUS‐agarose (#sc‐2003; Santa Cruz Biotechnology, Dallas, TX, USA, 1 : 50) was added and incubation at 4 °C for 6 h to bind and precipitate the antibodies which combined with chromatin DNA. Eventually, the immunoprecipitated DNA and Input DNA was de‐crosslinked by incubation at 65 °C and purified, and then, the two DNA samples were used to perform PCR and qRT‐PCR to test whether the antibodies can bind the promoter region of *BTN3A1*. The primers were listed in Table 3.

### Western blot

Cells and tissues were lysed with RIPA buffer, protein extracts were quantitated, subjected to 10% SDS/PAGE, electrophoresed, and transferred onto a PVDF membrane. The membrane was washed and incubated with the indicated primary and secondary antibodies after blocking with 5% nonfat milk in Tris‐buffered saline and then detected by electrochemiluminescence in Luminescent Image Analyzer LSA 4000 (GE, Fairfield, CO, USA). Antibodies used included rabbit anti‐β‐Actin (#ab8227; Abcam, Cambridge, UK; 1 : 5000) and rabbit anti‐BTN3A1 (#25221‐1‐AP; Proteintech; 1 : 500) antibodies.

### Statistical analysis

The data generated in Oncomine were displayed with *P* values, fold changes, and ranks. Survival curves were generated by the Kaplan–Meier plots and GEPIA. The results of Kaplan–Meier plots and GEPIA were displayed with hazard ratio and *P* values from a log‐rank test. The correlation of gene expression was evaluated by Pearson's correlation. The strength of the correlation was determined using the following guide for the absolute value: 0.00–0.29 ‘weak’, 0.30–0.49 ‘moderate’, 0.50–0.79 ‘strong’, 0.80–1.0 ‘very strong’. *P* values < 0.05 were considered statistically significant.

## Results

### The mRNA expression levels of *BTN3A1* in different types of human cancers

**Fig. 1:**
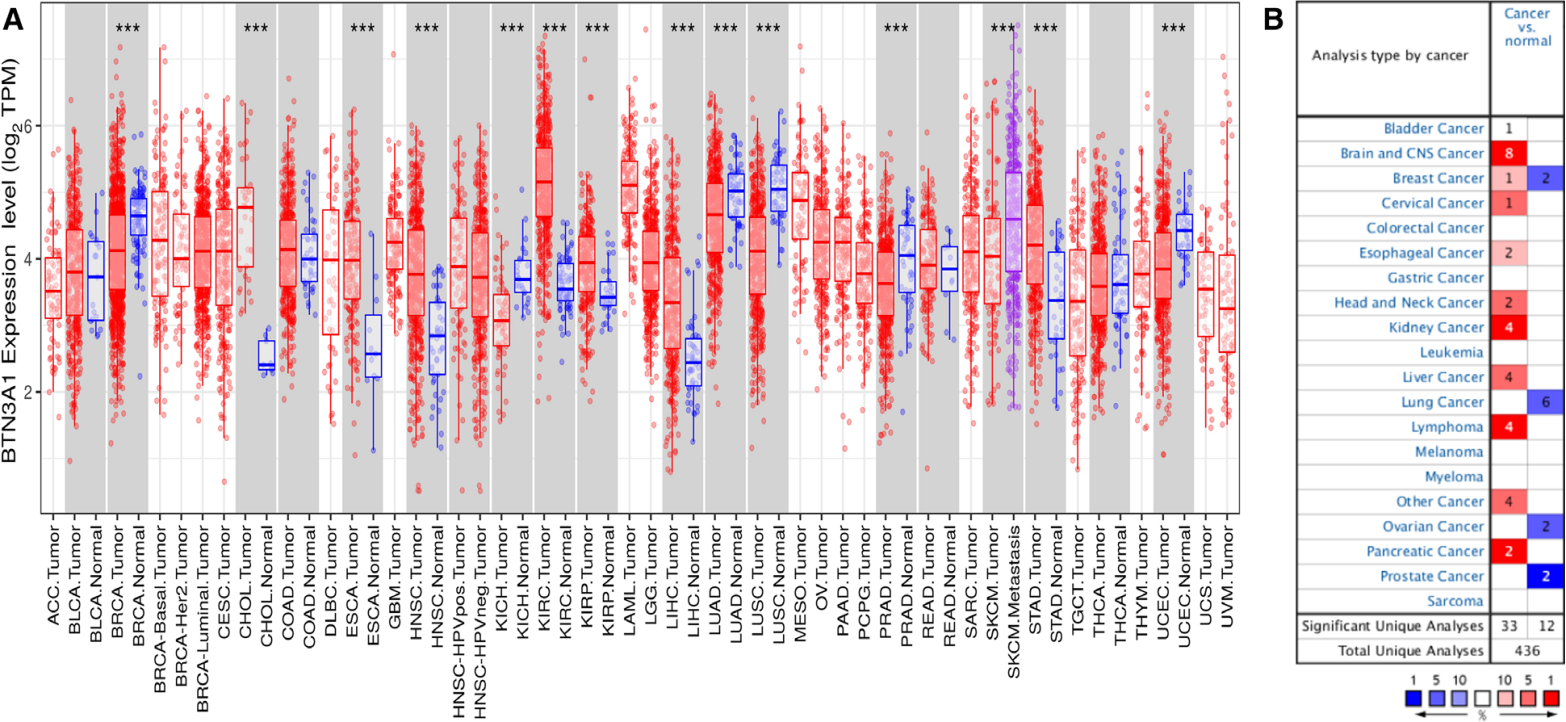
*BTN3A1* expression levels in different types of human cancers. (A) *BTN3A1* expression levels in different types of cancers. The expression of *BTN3A1* was determined by analyzing TIMER database containing TCGA datasets (***P < 0.001). (B) The expression level of *BTN3A1* in different cancers as compared to that in normal tissues by using the Oncomine datasets. The threshold was determined according to the following values: *P* value of 0.001, fold change of 1.5, and gene ranking of all.

The expression of *BTN3A1* in tumor and adjacent normal tissues was analyzed in TIMER database containing TCGA data, and the results showed that *BTN3A1* expression was significantly higher in tumor samples of cholangiocarcinoma (CHOL), esophageal carcinoma (ESCA), head and neck squamous cell carcinoma (HNSC), kidney renal clear cell carcinoma (KIRC), kidney renal papillary cell carcinoma (KIRP), liver hepatocellular carcinoma (LIHC), and stomach adenocarcinoma (STAD), compared to their counterpart normal tissue controls (Fig. 1A). In contrast, *BTN3A1* expression was significantly lower in breast cancer (BRCA), kidney chromophobe (KICH), lung adenocarcinoma (LUAD), lung squamous cell carcinoma (LUSC), prostate adenocarcinoma (PRAD), and uterine corpus endometrial carcinoma (UCEC), in comparison with that in counterpart normal tissue controls (Fig. 1A). In Oncomine database, the expression of *BTN3A1* was upregulated in tumor tissues than in normal tissue controls of patients with cancers of brain, cervical, esophageal, head and neck, kidney, and liver, and was downregulated in tumor samples of patients with cancers of breast, lung, ovarian, and prostate (Fig. 1B).

### Prognostic potential of *BTN3A1* in human cancers

**Fig. 2:**
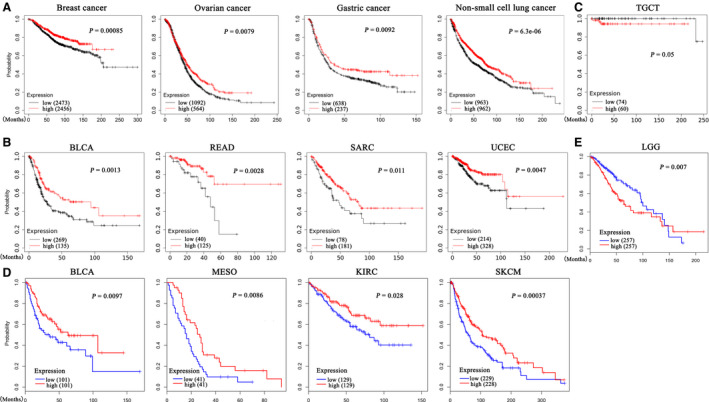
Overall survival of patients with different *BTN3A1* expression levels in multiple cancer types. (A) High expression of *BTN3A1* in breast cancer, ovarian cancer, gastric cancer, and NSCLC is associated with better prognosis. (B, C) Higher expression of *BTN3A1* in BLCA, READ, SARC, and UCEC (B) and lower expression of BTN3A1 in THYM (C) are related to better outcome in Kaplan–Meier plotter based on RNA‐seq data. (D, E) Higher expression of *BTN3A1* in BLCA, MESO, KIRC, and SKCM (D) and low expression in LGG (E) are associated with longer survival in GEPIA database.

We employed the Kaplan–Meier plotter database to evaluate the relationship between *BTN3A1* expression level and patient outcome in multiple cancer types. In datasets derived from the Affymetrix gene chips (Fig. 2A), higher expression of *BTN3A1* was associated with better prognosis in breast cancer, ovarian cancer, gastric cancer, and NSCLC. In datasets based on RNA‐seq (Fig. 2B), higher expression of *BTN3A1* was associated with better prognosis in bladder urothelial carcinoma (BLCA), rectum adenocarcinoma (READ), sarcoma (SARC), and UCEC. However, higher expression of *BTN3A1* indicated poor prognosis in patients with testicular germ cell tumors (TGCT) (Fig. 2C). In GEPIA datasets, higher *BTN3A1* expression was associated with longer overall survival in patients with BLCA, mesothelioma (MESO), KIRC, and skin cutaneous melanoma (SKCM) (Fig. 2D), whereas higher *BTN3A1* expression was associated with worse prognosis in patients with brain lower grade glioma (LGG) (Fig. 2E).

### Variations of *BTN3A1* in human cancers

**Fig. 3:**
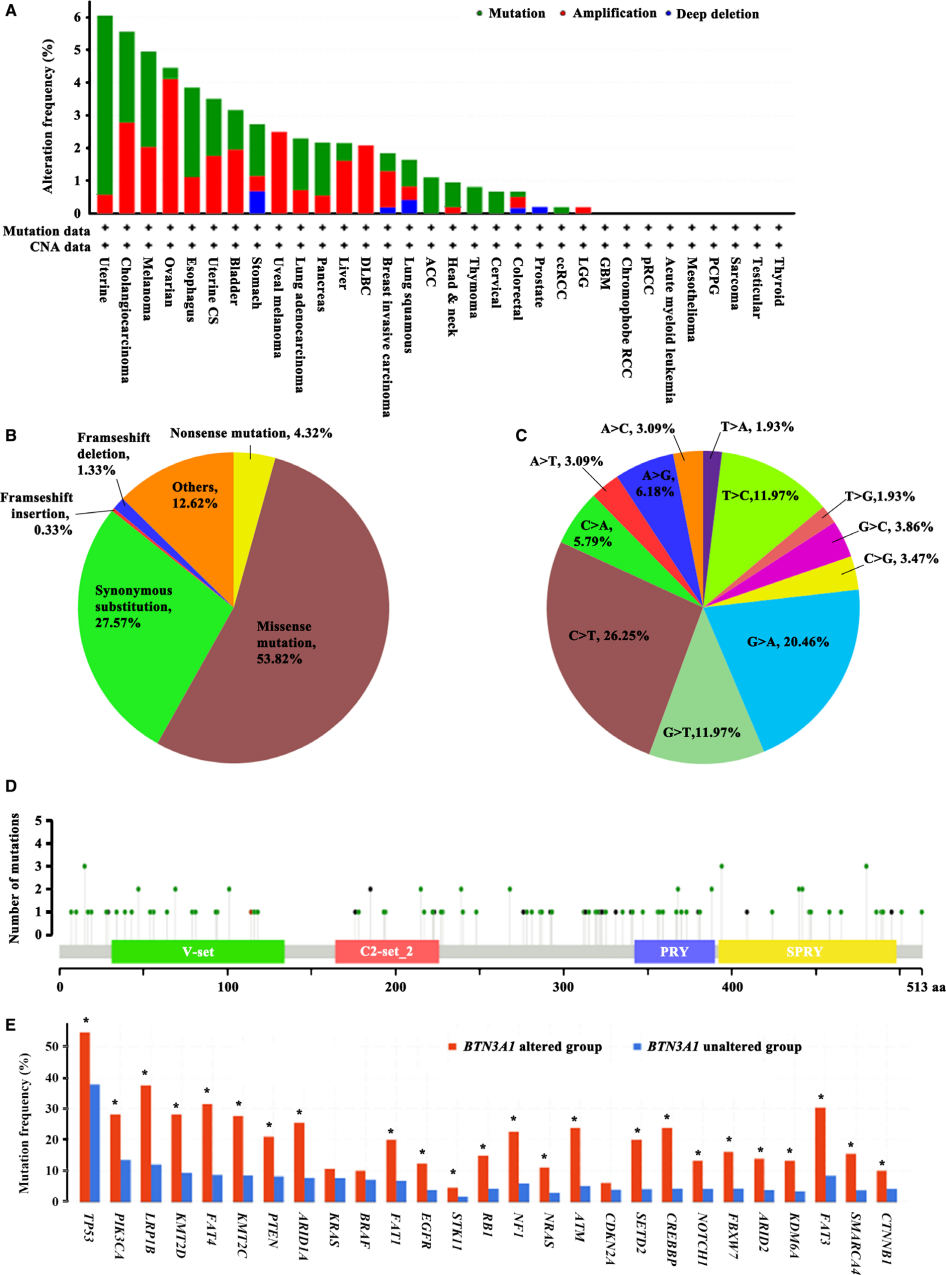
Mutations of *BTN3A1* in human cancers. (A) The mutation frequencies of *BTN3A1* in different cancers in cBioportal database. CNA, copy number alteration. (B) The mutation types of *BTN3A1* in cancers. (C) Nucleotide substitutions of *BTN3A1* in cancers. (D) The number of mutations and the affected amino acids of *BTN3A1* in cancers. (E) The frequency of driver mutations in patients of *BTN3A1* altered and unaltered groups (*P < 0.05).

The cBioportal was used to investigate the mutations of this gene, and the results showed that *BTN3A1* was mutated in cancers of uterine, CHOL, melanoma, and others (Fig. 3A). A total of 188 (1.72%) somatic mutations were seen in 10 953 patients with different types of cancers. The mutation frequency reached 32/529 (6.05%) in UCEC (Fig. 3A). Among the 383 *BTN3A1* nucleotide substitutions, 175 (58.14%) ones were nonsense or missense mutations (Fig. 3B). The C:G>T:A mutations were the most frequently detected nucleotide substitutions (46.71%) (Fig. 3C). The mutations were distributed throughout the entire gene (Fig. 3D), which is characteristic of mutations in potential tumor suppressors. The potential relationship between *BTN3A1* mutation and the well‐known driver mutations was analyzed, and the results showed that patients with mutant *BTN3A1* had higher mutation frequency in 24 genes including *TP53*, *PTEN*, *EGFR*, *STK11*, and others, than patients with wild type *BTN3A1* (Fig. 3E).

### *BTN3A1* correlated signal pathways in BRCA, LUAD, and LUSC

**Fig. 4:**
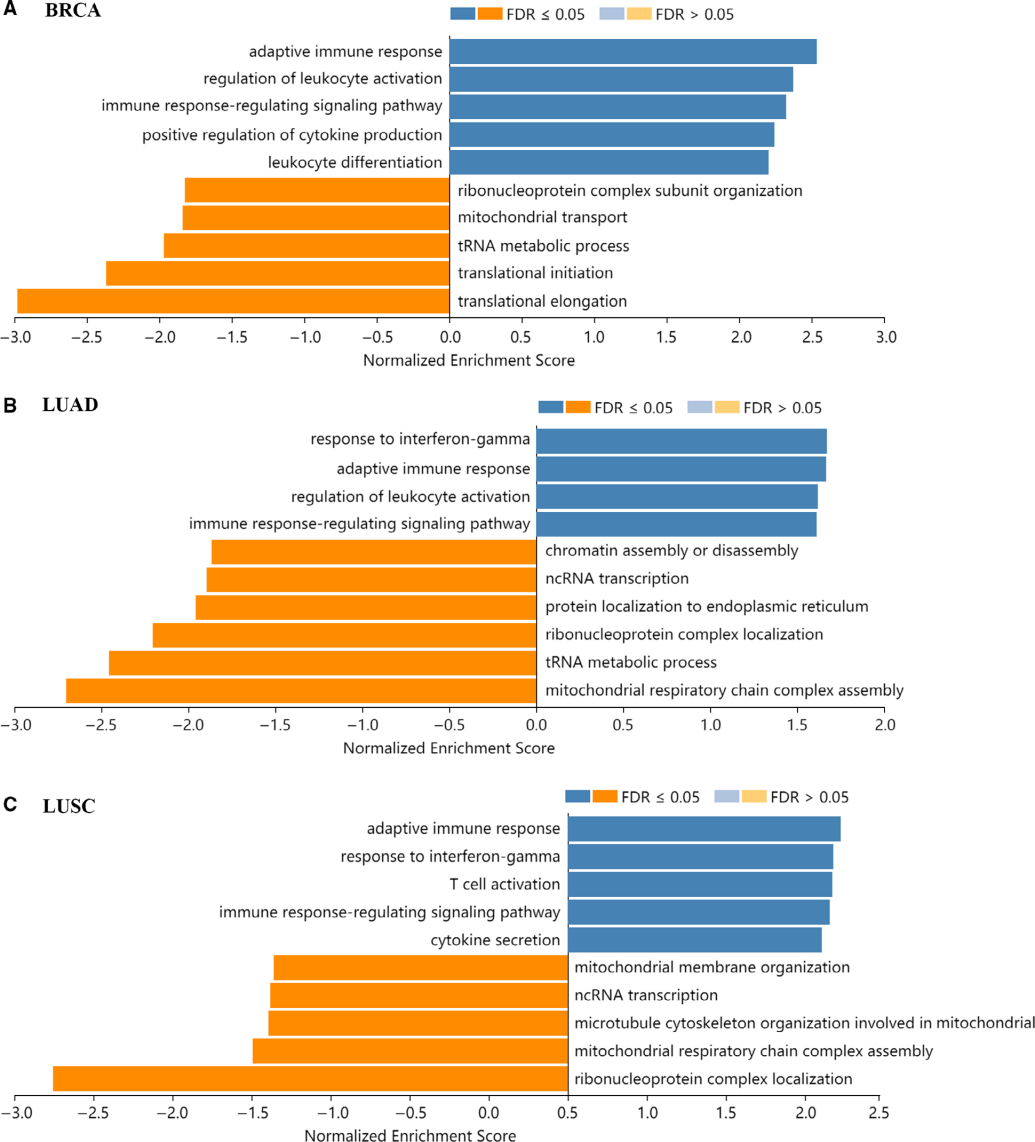
Significantly enriched GO Biological processes in correlation with *BTN3A1* expression in BRCAs (A), LUADs (B) and LUSCs (C). The *x*‐axis represents the normalized enrichment score, while the *y*‐axis represents the term of GO. FDR, false discovery rate.

Gene Ontology biological process for the GSEA of the Linkedomics database was employed to analyze the signal pathways that may be in correlation with *BTN3A1* expression during carcinogenesis. The results showed that pathways involving adaptive immune response and immune response‐regulating signaling were significantly enriched and were positively correlated with *BTN3A1* expression levels in BRCA, LUAD, and LUSC (Fig. 4A–C). Other immune‐related pathways, such as response to interferon gamma, regulation of leukocyte activation, and positive regulation of cytokine production, also appeared in the GSEA dataset (Fig. 4A–C).

### *BTN3A1* is correlated with immune infiltration and immune checkpoint expression

**Fig. 5:**
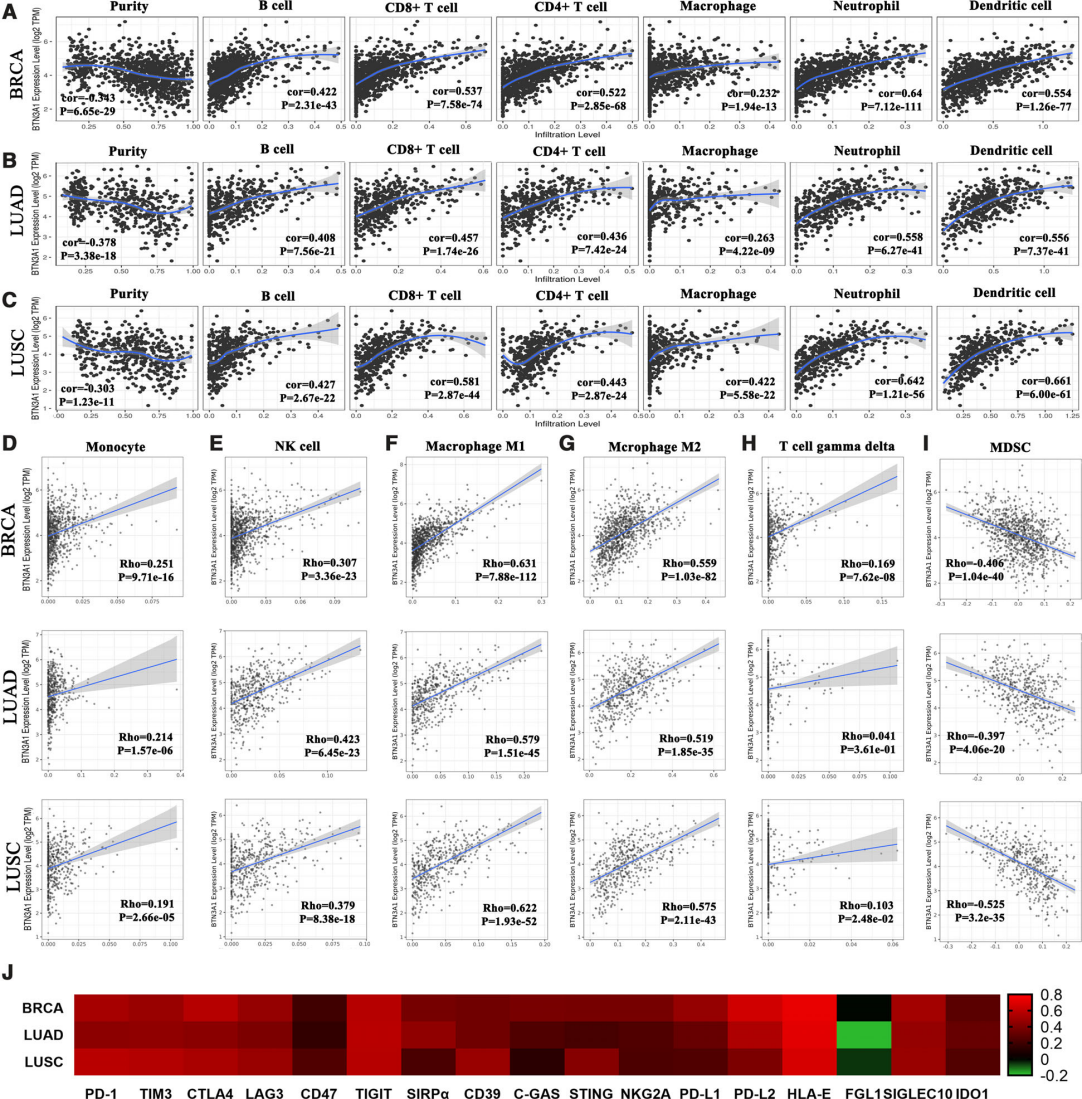
Correlation of *BTN3A1* expression with immune infiltration level in BRCAs, LUADs, and LUSCs. (A–C) *BTN3A1* expression is negatively correlated with tumor purity and is positively correlated with infiltrating levels of B cells, CD8^+^ T cells, CD4^+^ T cells, macrophages, neutrophils, and dendritic cells in BRCAs (A), LUADs (B) and LUSCs (C). (D–G) *BTN3A1* expression has significant positive correlations with infiltrating levels of monocytes (D), NK cells (E), M1 macrophages (F), and M2 macrophages (G), and infiltrating levels of γδ T cells (H) in BRCAs, LUADs, and LUSCs. (I) *BTN3A1* expression has significant negative correlations with infiltrating levels of MDSCs in BRCAs, LUADs, and LUSCs. (J) Heat map of correlations between *BTN3A1* and immune checkpoint genes in BRCAs, LUADs, and LUSCs.

The tumor‐infiltrating lymphocytes are associated with prognosis and response rate of immunotherapy [30]. We analyzed the correlation between the expression levels of *BTN3A1* and the infiltration status of immune cell in BRCA, LUAD, and LUSC by using the TIMER database. The results showed that *BTN3A1* expression was negatively associated with tumor purity and positively correlated with infiltration levels of B cells, CD8^+^ T cells, CD4^+^ T cells, macrophages, neutrophils, and dendritic cells (Fig. 5A–C). Furthermore, the TIMER2.0 database was employed to determine whether other immunocyte infiltration levels were correlated with *BTN3A1* expression. In this database, higher infiltration levels of monocytes, NK cells, M1, and M2 macrophages, and γδ T cells were positively associated with increased expression of *BTN3A1* in BRCA, LUAD, and LUSC (Fig. 5D–H). The numbers of myeloid‐derived suppressor cells (MDSCs) in BRCA, LUAD, and LUSC were negatively associated with the *BTN3A1* expression (Fig. 5I). In addition, the correlation between the expression of *BTN3A1* and the typical immune checkpoints was explored, and the results showed that the expression levels of *PD‐1*, *TIM3*, *CTLA‐4*, *LAG3*, *TIGIT*, *CD39*, *STING*, *PD‐L1*, *PD‐L2*, *HLA‐E*, *SIGLEC10,* and *IDO1* were positively associated with *BTN3A1* expression levels (Fig. 5J). The above data suggested that *BTN3A1* may have a role in modulating the immune microenvironment of cancers.

### Validation of the expression of BTN3A1 in NSCLC and BRCA patient samples

**Fig. 6:**
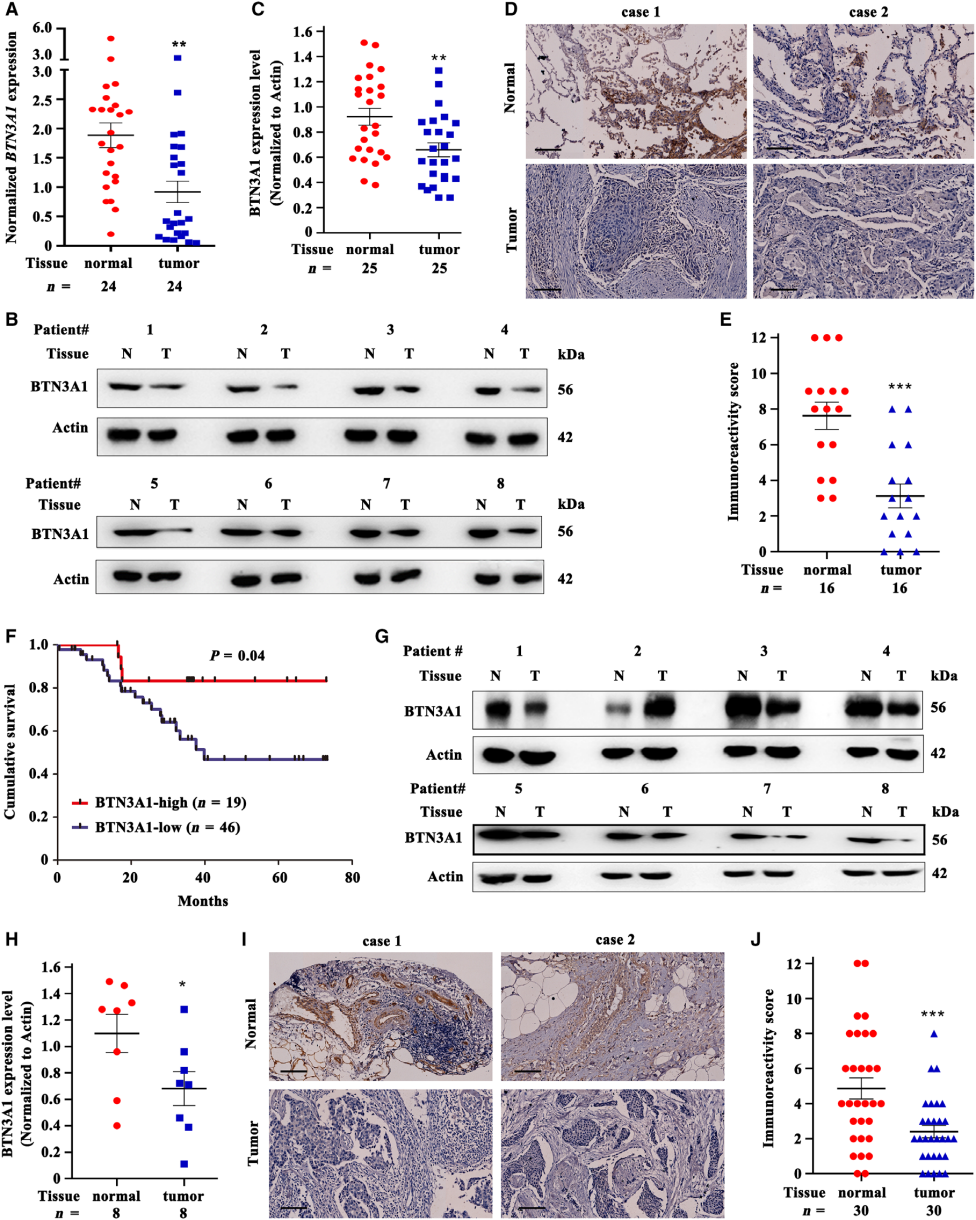
The expression of BTN3A1 in patients with NSCLC or BRCA. (A) The expression of *BTN3A1* in NSCLCs was detected by quantitative RT‐PCR. (B, C) The expression of BTN3A1 in NSCLCs was detected by western blot (B), and the western blot bands were assessed by densitometry analysis (C). (D, E) The expression of BTN3A1 in NSCLCs was tested by IHC assay (D), and the immunoreactivity score was calculated (E). (F) Overall survival of the 65 patients with NSCLC. (G, H) The expression of BTN3A1 in BRCAs was detected by western blot (G), and the western blot bands were assessed by densitometry analysis (H). (I, J) The expression of BTN3A1 in BRCAs was tested by IHC assay (I), and the immunoreactivity score was calculated (J). Scale bar = 100 μm; *P* values, Student's *t*‐test, *P < 0.05; **P < 0.01; ***P < 0.001. Error bars, SEM.

To validate the results obtained from the databases, we tested the expression of BTN3A1 in NSCLCs and BRCAs. Our results showed that BTN3A1 was lower in tumor samples than in counterpart normal controls in 46 (70.8%) of 65 NSCLCs (Table 1), detected by qPCR (Fig. 6A), western blot (Fig. 6B,C), and IHC (Fig. 6D,E). The expression level of BTN3A1 was inversely associated with clinical outcome of NSCLC patients (Fig. 6F). In BRCAs, BTN3A1 was lower in tumor samples than in counterpart normal controls in 31 (81.6%) of 38 patients' samples (Table 2), detected by western blot (Fig. 6G,H) and IHC (Fig. 6I,J) assays.

### *BTN3A1* is a target of transcription factor Spi‐1 proto‐oncogene

**Fig. 7:**
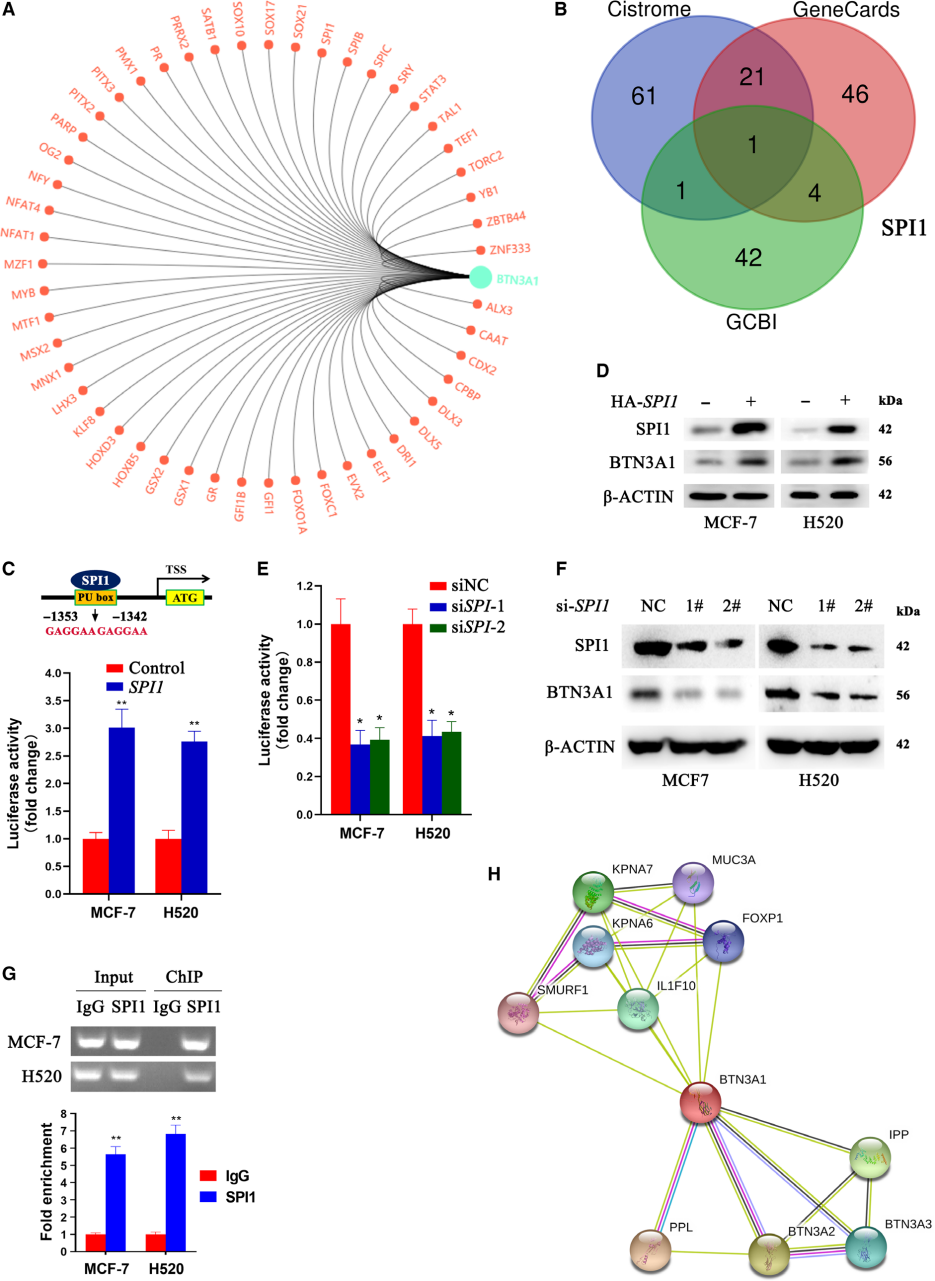
*BTN3A1* is a target gene of SPI1. (A) 48 transcription factors are predicted to be able to regulate *BTN3A1* in GCBI online database. (B) Cross‐referencing of the transcription factors in Cistrome data browser, GeneCards, and GCBI databases. (C) Luciferase assays was performed in MCF‐7 and H520 cells transfected with *BTN3A1* promoter‐driven luciferase and *SPI1* construct. Biologically independent replicates, *n* = 3. (D) Western blot assays using lysates of the cells transfected with SPI1 and indicated antibodies. (E) Luciferase assays were performed in MCF‐7 and H520 cells with *BTN3A1* promoter‐luciferase reporter construct and siRNAs. Biologically independent replicates, *n* = 3. (F) Western blot assays using lysates of the cells transfected with siRNAs and indicated antibodies. (G) ChIP assay was performed using SPI1‐precipitated DNA samples of MCF‐7 and H520 cells and primers for *BTN3A1*. The expression of *BTN3A1* was quantified by RT‐PCR (upper panel) and qPCR (lower panel). Biologically independent replicates, *n* = 3. (H) The correlation of *BTN3A1* and other proteins is analyzed in String database. *P* values, Student's *t*‐test, *P < 0.05; **P < 0.01. Error bars, SEM.

We investigated the transcription factors that may control *BTN3A1* expression by using the GCBI online software. A total of 48 transcription factors were identified (Fig. 7A). In Chip‐seq data from Cistrome and GeneCards, we showed that SPI1 (also known as PU.1) could bind to the promoter region of *BTN3A1* in these three databases (Fig. 7B). By analyzing the sequence of *BTN3A1* gene, we found two SPI1 binding sites (PU boxes), 5′‐GAGGAA‐3′, in −1353 to −1348 and −1347 to −1342 bp upstream of the transcription start site (TSS) (Fig. 7C, upper panel). In *in vitro* luciferase reporter assays, we showed that overexpression of SPI1 in MCF‐7 and H520 cells enhanced luciferase activity driven by *BTN3A1* promoter (Fig. 7C, lower panel). Ectopic expression of SPI1 upregulated BTN3A1 at protein level (Fig. 7D). In the two lines, silencing of *SPI1* by siRNAs significantly reduced *BTN3A1* promoter‐driven luciferase activity (Fig. 7E). Knockdown of *SPI1* also led to downregulation of BTN3A1 at protein level (Fig. 7F). ChIP assays were performed in MCF‐7 and H520 cells by using an anti‐BTN3A1 antibody or a control IgG. The results of RT‐PCR (Fig. 7G, upper panel) and qPCR (Fig. 7G, lower panel) confirmed that SPI1 was able to recruit *BTN3A1* promoter. The PPI network of BTN3A1 was constructed by mining the STRING database, and the top ten proteins most connected to BTN3A1 were PPL, BTN3A2, IPP, KPNA7, IL1F10, BTN3A3, KPNA6, FOXP1, MUC3A, and SMURF1 (Fig. 7H).

## Discussion

Butyrophilin belongs to Ig superfamily and contains both B7 and B30.2 domains. Most BTN family members are reported to play vital roles in regulating immunity. Among them, *BTN3A1* is widely expressed in immune cells and tumor cells and can regulate the immune response of T cells, especially the γδ T cells [12, 16]. In this study, we performed Pan‐cancer analysis to evaluate the role for *BTN3A1* to play in tumorigenesis and reported that there was a significant differential expression pattern of *BTN3A1* among the 33 cancer types. In patients with breast cancer, ovarian cancer, gastric cancer, NSCLC, BLCA, READ, SARC, UCEC, MESO, KIRC, and SKCM, higher expression of *BTN3A1* was associated with better prognosis; but in patients with TGCT and LGG, higher expression of *BTN3A1* was associated with worse prognosis. These results suggest that *BTN3A1* may play context‐dependent roles in different types of cancers; therefore, its activities in different cancer types should be carefully investigated in the future. Breast cancer and lung cancer represent the most commonly diagnosed cancer in female and male, respectively [31]. *BTN3A1* expression was downregulated in breast cancer and NSCLC, and was positively associated with clinical outcome of the patients. We showed that *BTN3A1* mutations are found in several cancers at the coding regions of each domain. In addition, the frequency of driver gene mutations in patients with mutant *BTN3A1* was significantly increased, suggesting that *BTN3A1* may play an important role in carcinogenesis.

BTN3A1 can activate gene expression during myeloid and B‐lymphoid cell development and has been reported to be expressed in a variety of immune cells and involved in a variety of immune regulatory processes [32]. We showed that *BTN3A1* was positively correlated with the infiltration of B cells, CD4^+^ T cells, CD8^+^ T cells, macrophages, dendritic cells, γδ T cells, and NK cells, and negatively correlated with the infiltration of MDSCs. BTN3A2, BTN3A3, FOXP1, and IL1F10, which are important for immune response [17, 33, 34], were included in the PPI network of BTN3A1. The expression of *BTN3A1* was significantly decreased in NSCLC and BRCA. In these cancer types, the co‐expressed genes of *BTN3A1* were mainly distributed in immune regulation‐related pathways, such as differentiation and activation of immune cells, cytokine secretion, and immune response. The downregulated BTN3A1 may be associated with the decrease of tumor‐infiltrating immune cells such as CD8^+^ T cells, dendritic cells, γδ T cells, and NK cells, leading to suppression of tumor inhibition effects and worse prognosis of patients. The results suggested that BTN3A1 may have a role in shaping the immunosuppressive tumor microenvironment and may sever as a tumor suppressor in breast cancer and NSCLC by promoting the invasion of innate and adaptive immune cells and inhibition of the invasion of MDSCs.

We screened for transcription factors that can regulate *BTN3A1* in Cistrome, GeneCards, and GCBI, and found that SPI1 was the only transcription factor in the three databases that may bind *BTN3A1* promoter. The regulatory effects of SPI1 on *BTN3A1* were confirmed by our ‘wet’ experiments. The expression of SPI1 in NSCLCs has been reported, and increased number of PU.1+ cells is correlated with favorable prognosis of adenocarcinoma and poor prognosis of squamous cell carcinoma [35]. PU.1 is also a major transcriptional activator of the tumor suppressor gene LIMD1 [36]. These results suggest that the SPI1‐BTN3A1 pathway may have an important role in lung carcinogenesis, but the effects of SPI1‐BTN3A1 on immune suppressive microenvironment and related mechanisms remain to be elucidated.

## Conflict of interest

The authors declare no conflict of interest.

## Author contributions

FL and G‐BZ conceived and designed the project; FL, CZ, S‐HG, F‐YY, and G‐ZW acquired the data; FL, G‐ZW, and HG analyzed and interpreted the data; FL, G‐BZ, and G‐ZW wrote the paper.

## Data Availability

All data generated or analyzed during this study are included in this published article or are available from the corresponding author on reasonable request.
